# Governance Perspectives on Genetically Modified Animals for Agriculture and Aquaculture: Challenges for the Assessment of Environmental Risks and Broader Societal Concerns

**DOI:** 10.3390/ani15182731

**Published:** 2025-09-18

**Authors:** Marion Dolezel, Michael F. Eckerstorfer, Marianne Miklau, Anita Greiter, Andreas Heissenberger, Stefan Hörtenhuber, Sarah-Joe Burn, Werner Zollitsch, Karen Kastenhofer, Kristin Hagen, Margret Engelhard

**Affiliations:** 1 Land Use & Biosafety Unit, Umweltbundesamt—Environment Agency Austria, Spittelauer Laende 5, 1090 Vienna, Austria; michael.eckerstorfer@umweltbundesamt.at (M.F.E.); marianne.miklau@umweltbundesamt.at (M.M.); anita.greiter@umweltbundesamt.at (A.G.); andreas.heissenberger@umweltbundesamt.at (A.H.); 2 Department of Agricultural Sciences, BOKU University, Gregor-Mendel-Straße 33, 1180 Vienna, Austria; stefan.hoertenhuber@boku.ac.at (S.H.); sarah.burn@boku.ac.at (S.-J.B.); werner.zollitsch@boku.ac.at (W.Z.); 3 Institute of Technology Assessment, Austrian Academy of Sciences, Bäckerstraße 13, 1010 Vienna, Austria; karen.kastenhofer@oeaw.ac.at; 4 Division Assessment Synthetic Biology, Enforcement Genetic Engineering Act, Federal Agency for Nature Conservation, Konstantinstr. 110, 53179 Bonn, Germany; kristin.hagen@bfn.de (K.H.); margret.engelhard@bfn.de (M.E.)

**Keywords:** genetic modification, genome editing, new genomic techniques, environmental risk assessment, sustainability assessment, technology assessment, livestock, animal welfare, risk governance

## Abstract

Biotechnological applications in animals are increasingly developed for use in agriculture and aquaculture to tackle breeding challenges in animal production. By examining two case studies of genetically modified (GM) farmed animals relevant to the European Union, slick-haired cattle and growth-enhanced carp, we highlight the challenges for environmental risk assessment and discuss available assessment approaches to address broader societal concerns. We find that the existing guidance for environmental risk assessment of GM animals available in the European Union faces several challenges. Assessing risks of GM animals in agriculture and aquaculture requires consideration of the farming systems of these animals. In addition, we find that there is a lack of guidance and practical implementation to address wider issues, including cultural, societal, ethical, and socio-economic issues, as well as animal health and welfare issues, related to GM farmed animals. We propose using existing assessment frameworks to address the sustainability of GM farmed animals beyond environmental risk assessment. Sustainability assessment approaches should also address potential farm-level sustainability claims of GM animal applications. We note that issues related to animal health and welfare are cross-disciplinary topics that require special attention when commercializing GM farmed animals. We recommend developing a comprehensive framework, including risk assessment, sustainability assessment, and technology assessment, that will enable policymakers to better anticipate and address the societal, legal, ethical, and governance issues associated with emerging biotechnologies in farmed animals.

## Simple Summary

Advances in biotechnology enable genetic modification, including genome editing of animals intended for the food chain. Innovative approaches increasingly include strategies to genetically modify traits of interest for animal production in agriculture and aquaculture. We discuss adequate governance approaches to these innovations by presenting an overview of established methods and expert opinions. We find that, while risk assessment guidelines are available to identify and assess environmental risks, environmental risk assessment still faces considerable challenges, and governance concepts and methodologies to address broader societal issues raised in the expert literature must still be developed.

## 1. Introduction

In recent years, new biotechnological tools have changed not only plant but also animal breeding. Editing the genomes of economically important animals used for human food production, such as terrestrial and aquatic farmed animals (e.g., fish) has become a promising field of research. Similar as for crop plants, new genomic techniques (NGTs) developed for the targeted modification and editing of genes, such as zinc-finger nucleases (ZFN), transcription activator-like effector nucleases (TALEN), or, more recently, clustered regularly interspaced palindromic repeats (CRISPR) and the CRISPR-associated protein 9 (Cas9), are increasingly applied to tackle breeding challenges relevant for animal production. The possibility of more precise modification of animal genomes by use of NGTs, combined with in vitro methods to produce genetically modified (GM), including genome edited (GE) animals by zygote manipulation or somatic cell nuclear transfer (i.e., animal cloning) [1], enables the targeted modification of traits in farmed animals which are difficult to achieve by conventional breeding strategies. The resulting biotechnological applications, also referred to as “breeding by design” or “Livestock 2.0” [2,3], focus on traits relevant for modern husbandry of farmed animals [2,4,5,6,7,8,9].

In the European Union (EU), GM animals—developed by either classical transgenesis or by one of the NGTs—have, so far, not been authorized for food or feed use. In 2018, the European Food Safety Authority EFSA, the EU’s central risk assessment body for the agri-food chain, received a mandate from the European Commission to develop an opinion on genetically modified organisms (GMOs) developed through synthetic biology and their implications for risk assessment methodologies. Part of this mandate is the consideration of new developments in biotechnology applied to animals, including synthetic biology and NGTs. A scientific opinion is due to be published by the EFSA in 2025. The aim is to identify potential novel hazards and risks from the new developments in biotechnology applied to animals and to assess the adequacy of the risk assessment guidance documents currently available and relevant for GM animals [10,11]. As part of this scientific opinion, a horizon scan was carried out on behalf of the EFSA to gather knowledge on known cases of animals, for food and feed purposes and for agricultural uses, being obtained by new developments in biotechnology [12]. The report identified the most important animal species, traits, and techniques applied to modify the genome of farmed animals [12]. Altogether, 113 mammal, 53 aquatic, 15 poultry, and 7 insect species were reported, with CRISPR/Cas9 being the most frequently used biotechnological tool. The modifications focus on traits like yield, altered reproduction, biotic stress tolerance, allergenicity, color, quality, or animal welfare [12]. Worldwide, a few GM or GE animals have already been commercialized in at least one country, specifically, growth-enhanced fish (salmon, sea bream, and tiger pufferfish), an anti-allergenic pig, growth-enhanced cattle, and cattle intended to exhibit increased thermotolerance [12].

Another recent horizon scan of environmental applications of GM terrestrial animals, including GE animals, showed that research efforts are underway on a range of farmed animals, including cattle, goat, sheep, pig, horse, rabbit, chicken and quail [9]. The modified traits predominantly aim to increase the efficiency and productivity of farm-level production, such as improved disease resistance, increased performance (e.g., muscle growth and milk yield), change in reproductive traits (e.g., sex reversal and sterility), or mitigating animal welfare issues, e.g., polledness and the genetic absence of horns [9,13]. Traits relevant for product quality (e.g., milk, meat, or wool quality) are by far less frequently targeted [5,9,12]. With respect to fish, Miklau et al. [9] identified a wide range of modified fish species with enhanced aquaculture performance traits, primarily enhanced muscle growth.

A notable feature with the emergence of biotechnology applications of farmed animals is the discussion of their potential benefits and contributions to food security and sustainable production by developers, regulators, and scientists [14,15,16,17,18]. The application of biotechnology in animal breeding is often put forward as a means to improve animal welfare and resilience in animal farming, facilitate management of pests and diseases, and provide economic benefits by increased productivity [2,4]. In addition, biotechnological approaches claim to provide new solutions to environmental issues of animal farming, such as the reduction in environmental phosphorous load [19] or the preservation of wild fish stocks against escaped GM farmed fish by rendering them genetically sterile [20]. Often, the reasons for applying genome editing in farmed animals are based on economic incentives rather than on animal-related concerns such as welfare or moral status [21]. In combination with the required reproductive methodologies, the development phase will typically be costly in terms of animal welfare [22,23]. Compared to classical breeding strategies, the efficiency of the new biotechnological tools to achieve improved traits in animals and, consequently, increased productivity, is one of the main reasons for the use of genome editing in animal breeding [5,21].

The purpose of this review is to outline relevant issues of emerging biotechnology applications in farmed animals for agriculture and aquaculture that are relevant for the EU. It is based on the results of a horizon-scanning activity to identify potential future GM applications with environmental relevance developed for terrestrial animals (excluding insects and gene drives), fish, microalgae, and micro-organisms [9]. We discuss two different case studies of GM farmed animals with different commercial importance in the EU—the slick-haired cattle with intended thermotolerance and the growth-enhanced common carp. We chose these examples based on the results of a recent horizon scan [9]. We aimed to include one land animal and one aquatic species. Both case studies have been developed by the use of NGTs, specifically CRISPR/Cas9. The application of these tools, in living organisms intended for environmental release, is subject to the current regulations for GMOs in the EU, specifically Directive 2001/18/EC and Regulation (EC) No. 1829/2003. Therefore, they are referred to as GMOs in this article.

We first discuss knowledge gaps for the two case studies as well as the challenges for environmental risk assessment (ERA), specifically with respect to the comparative approach and the assessment of risks to the environment, as well as animal health and welfare, based on the existing guidance documents developed for ERA of GM animals issued by the EFSA [10,11]. We then outline broader societal concerns and sustainability issues relevant for the two applications of GM farmed animals. Such considerations, which go beyond environmental risk assessments, are becoming increasingly relevant with emerging biotechnology applications of animals developed for use in agriculture and aquaculture. They are addressed by established approaches such as sustainability assessment (or sustainability analysis, SA) and technology assessment (TA). Both of these approaches aim towards providing a comprehensive understanding of all social, economic, and ecological aspects that are critical for sustainable development and informing decision-makers, in a quest to make societies more sustainable. Compared to the ERA, they are much more integrative (both, regarding interdisciplinarity and transdisciplinarity), but they are also less formalized, with TA probably being the least formalized and the most process-oriented. TA thus often serves to ‘open-up’ critical debates rather than ‘close down’ opinion making, introducing a wide variety of potentially critical issues and considering various policy options [24].

In the later sections of this paper, we analyze whether sustainability and technology assessment frameworks and expert opinions are available to address the empirical cases at hand. In our concluding section, we integrate our findings for ERA, SA, and TA of GM animals for aquiculture and agriculture.

## 2. Examples of Emerging Applications of GM Animals for Agriculture and Aquaculture

### 2.1. Slick-Haired Cattle

In cattle (*Bos taurus*), slick-haired individuals, also referred to as PRLR-SLICK cattle, were developed within the Angus breed using a genome-editing approach [4,25]. Information on the GM cattle is provided by Porto-Neto et al. [26] and Hansen [27]. The prolactin-receptor (PRLR) gene was disrupted by using CRISPR/Cas9. The deletion introduced a premature stop codon, causing a frameshift mutation and leading to a truncated form of the PRL (prolactin) receptor protein. Slick-haired cattle have shorter and sleeker hair and an improved thermoregulatory ability due to the reduced insulation of their hair coat, which facilitates heat loss from their skin. The phenotypic effect is caused by changes in the cell-signaling properties of the PRL receptor in the hair follicle [27].

The slick-hair trait occurs naturally in tropical cattle breeds such as Senepol cattle and Criollo breeds in Central and South America [27,28]. Different genetic variants of (non-GM) slick-haired cattle with up to six different mutations have been described [26,27,29]. All these natural variants carry mutations in a specific genomic region of the *PRLR* gene, and all mutants confer the short hair phenotype. The targeted *PRLR* gene has been described as a single dominant gene on bovine chromosome 20 [28,30] and no phenotypic differences between homozygous and heterozygous individuals have been reported [30]. The slick-hair mutation has also been introduced, by classical breeding methods, into other breeds such as Holstein [4,30]. In addition to the differences in hair coat, the animals show increased sweating, as well as lower respiration rates, and rectal and vaginal temperatures [30,31,32]. Under calving conditions in the summer, the reduction in their milk yield is less pronounced due to the lower energy requirement for thermoregulation and the minimal effect on feed intake [30,33].

The slick-haired GM cattle have undergone regulatory evaluations in at least three countries, the US, Brazil and Argentina [12]. In the US, the regulatory authority, the Food and Drug Administration (FDA), has published a risk assessment summary for slick-haired cattle [25]. The cattle were subject to an enforcement discretion in 2022 by the FDA, which made a low-risk determination for the marketing of products from slick-haired cattle based on a food risk assessment [5,25]. In May 2024, the FDA published two draft guidance documents for the approval process and the regulatory approach for heritable intentional genomic alterations in animals [34,35]. Therein, the US authority clarified that it did not expect specific risks for the described genomic alterations in the respective slick-haired cattle, as these lead to traits which are also found in conventionally bred species and, for which, a history of safe use is available [34]. In Brazil, the slick-haired cattle have been determined to be non-GMO and, therefore, not regulated (technical opinion of the respective authority; see [12,36]). In Argentina, GM cattle with slick-hair trait without vector DNA have been determined to be conventionally bred animals [14].

### 2.2. Growth-Enhanced Fish Including Common Carp

The first growth-enhanced GM fish, a transgenic Atlantic salmon commercialized as AquAdvantage salmon™ (AquaBounty Technologies, Inc., Harvard, MA, USA), received approval by Canadian regulatory authorities, Health Canada and Environment Canada, for food, feed, and environmental safety in 2013 [37,38]. Both authorities approved the use of the GM salmon for commercial purposes in two specific production facilities under strictly controlled conditions, including physical confinement. In 2024, the company AquaBounty Technologies announced a cease in salmon farming operations in Canada, including in the production of GM Atlantic salmon [39]. In 2015, the US Food and Drug Administration (FDA) approved the same GM salmon as safe to eat [40]. In 2021, the Brazilian National Biosafety Technical Commission (CTNBio) also approved growth-enhanced GM salmon [12]. Recently, Japan was the first country to approve the marketing of two GE growth-enhanced fish species, the Red Sea bream and tiger pufferfish, without any risk assessment. Both GM fish were developed by using CRISPR/Cas9. In Argentina and Brazil, the respective authorities assessed a growth-enhanced GM Nile tilapia [12]. No risk assessments were conducted, since these applications do not fall under the biosafety laws of the respective countries.

The earliest reports of genetic modification of common carp (*Cyprinus carpio*), specifically referring to the transgenic expression of a growth hormone gene, date back to the 1990s and were reported from Hungary [41]. In addition, Chinese researchers developed a growth-enhanced common carp by use of transgenesis (see overview in [42]). In a recent proof-of-concept study, Zhong et al. [43] used two different site-specific genome-editing tools (TALEN and CRISPR/Cas9) to modify genes responsible for muscle development with the aim to achieve a growth-enhanced common carp. The modified *mstnba* gene codes for a growth factor (myostatin), which negatively regulates muscle growth. The knockout mutations in the *mstnba* gene caused frameshift mutations resulting in a truncated peptide, leading to an impaired signaling pathway for myostatin in skeletal muscles [43]. The mutations in the GM fish were mosaic, i.e., located in different tissues and organs, and were also detected in their testes, indicating that they could be transmitted to subsequent generations. Phenotypically, the GM fish had more muscle cells and fibers, as well as increased muscle fiber size, leading to significantly increased fish weight and body size.

## 3. Knowledge Gaps Regarding GM Animals for Agriculture and Aquaculture

### 3.1. Slick-Haired Cattle

So far, knowledge about slick-haired cattle, with regard to the functionality of the GM trait and potentially unintended effects, is very limited. The developers confirmed the intended phenotype, i.e., the slick-hair trait with shorter and sleeker hair, in two modified individuals. The observed changes in the hair coat are considered to reduce the adverse effects of increased ambient air temperature on cattle health and welfare as well as on productivity, e.g., in hot climates [5,25]. However, the phenotypic functionality of the GM trait under relevant conditions (i.e., heat) was not assessed by the developers, but rather inferred from the similarity of the GM genotype to naturally occurring genetic variants [25]. In addition, in the two founder calves, unintended molecular changes that related to a duplication and indels in intergenic regions were identified by the molecular characterization and a whole genome sequencing approach [25]. The US authority concluded that no differences in development or animal health were observed between slick-haired cattle and its conventional comparator, based on the molecular characterization and the (short) observation of the two modified animals, but may revisit its decision depending on the availability of new information [25,34,35].

Potential unintended phenotypic changes in GM cattle may constitute potential hazards for animal health or welfare. The prolactin receptor gene is a pleiotropic gene, and, therefore, the modified and truncated prolactin protein may also affect other physiological pathways and functions, but details still need to be clarified. Prolactin expression has been documented in a range of body organs and tissues and has relevance for more than 300 biological functions [44,45]. There is evidence for the effects of the slick mutation on thermoregulation-unrelated physiological pathways, such as changes in liver gene expression [46]. Also, negative correlations between thermotolerance and milk yield or fertility have been described [27]. In addition, the slick phenotype can also affect milk composition and lactation [30,31].

Thermoregulation in cattle is a complex trait. Increased thermotolerance of cattle breeds evolved under hot and arid climatic conditions. Certain subtropical and tropical cattle breeds can, therefore, better regulate body temperatures and thereby tolerate high ambient air temperatures. For example, breeds of the zebu species (*Bos indicus*) have a range of physiological and morphological adaptations to cope with heat stress, such as a greater skin surface to mass ratio, a different pigmentation, and larger sweat glands. For breeds of *Bos taurus* that evolved under temperate conditions, such functional evolutionary adaptations to heat are absent, and they are better adapted to cope with cold stress [47]. In recent decades, the breeding efforts in cattle for increased milk production and growth increased the metabolic heat production and added more pressure on physiological regulation, which resulted in additional internal heat stress in these animals [48]. Common breeding indices show that heat tolerance has decreased with increasing performance in cattle [49]. The physiological thermoregulation mechanisms, which are needed by the animals to cope with heat stress, lead to reduced productivity due to reduced feed intake and higher energy requirements [50].

It is known that, in addition to the *PRLR* gene, other genes are also involved in thermotolerance in cattle [27,30]. Porto-Neto et al. [26] described several mutations of the *PRLR* gene in slick-haired cattle. However, some of the animals did not have any of these PRLR mutations but, nevertheless, exhibited a slick-hair phenotype, suggesting that this phenotype, in cattle, is also possible without the mutations in the *PRLR* gene [30]. In addition, the increased sweating observed in conventional slick-haired cattle is not only due to the differences in their hair coat but other physiological processes, and, so far, it is unclear whether these are affected by the mutations in the *PRLR* gene [27].

In addition, the response of GM cattle under cold conditions is so far not understood. Heat stress and exposure to solar radiation, but also cold stress, can induce changes in behavioral patterns in cattle, such as standing time, and can add to animal welfare problems [32,51,52]. Cold-sensitivity trials considering typical winter conditions of conventionally bred slick-haired cattle are underway in New Zealand, but results are still preliminary [53].

### 3.2. Growth-Enhanced Common Carp

The proof-of-concept study on GM growth-enhanced carp provided by Zhong et al. [43] focused on the confirmation of the functionality of targeted mutagenesis in this fish species by applying specific genome-editing approaches. The authors reported the intended increase in body weight and length in the mutant fish lines, but details on the phenotypic changes in the GM fish are lacking, particularly for a time period longer than three months. So far, there is little information on other phenotypic changes in the growth-enhanced carp, but there is considerable knowledge gained from other approaches, specifically, in transgenic carp or salmon or physiologically growth-enhanced fish, i.e., by intraperitoneally implanting a growth hormone [54,55,56,57]. Information on the productivity gains of other *mstn*-mutant, growth-enhanced GM fish varies considerably and includes increased body length, weight, feed intake, growth rate, or higher feed efficiency [57,58,59,60]. Transgenic growth-enhanced carp consumed almost double as much food as non-GM fish throughout the year [58,61,62]. Observed changes in body shape included increased body thickness or reduced body length [63,64,65].

In addition, other physiological, developmental, or behavioral traits can also be affected in growth-enhanced fish [54,55,66]. For example, the changed swimming abilities of GM fish were noticed due to the distinctive morphological changes in growth-enhanced carp [66]. Some of these observed unintended phenotypic effects are either directly related to the increased growth rate (e.g., feed intake), or are considered pleiotropic effects (e.g., weaker immune responses). The various phenotypic responses observed in growth-enhanced, transgenic GM fish are considered to be either a direct consequence of the rapid growth or an indirect consequence of the genetic modification of the specific metabolic pathway [54,55]. Nevertheless, the different mechanisms between the transgenic and the genome-editing approach to achieve growth-enhancement have to be taken into consideration. In transgenic carp, growth-enhancement has been achieved by the overexpression of a growth hormone that is known to affect multiple metabolic pathways and many phenotypic traits. In contrast, the knowledge about the role of myostatin genes in fish physiology and development is so far limited. Myostatin is a member of the growth factor β-family, which is expressed in skeletal muscle cells (overview in [67]). In GM mice, targeted deletions in the *mstn* gene encoding myostatin lead to increased muscle fiber size, and excess levels of myostatin caused muscle wasting [68]. Common carp have four *mstn* genes (*mstnba*, *mstnbb*, *mstnaa*, and *mstnab*), which are expressed in different organs. Their expression levels depend, however, on the specific type of gene, with the modified mstnba being expressed in most tissues and organs [43].

## 4. Challenges for ERA of GM Animals for Agriculture and Aquaculture in the EU

Experience with the market authorization of GM farmed animals in the EU is limited. So far, no GM animals or GM animal products have been approved for placement on the market in the EU, either for release into the environment or food and feed use (i.e., under the regulations of Directive 2001/18/EC or Regulation No. (EC) 1829/2003). Two guidance documents for the risk assessment of GM animals, including the assessment of food and feed risks and environmental risks, have been published by the EFSA in 2012 and 2013 [10,11]. These documents also contain guidance for the molecular characterization and the comparative approach, which are the starting points for ERA [10,11]. The ERA of GM animals follows a common methodology, with information requirements for different areas of environmental risks as outlined in the EFSA Panel on Genetically Modified Organisms [11] (see also Table 1). On a case-by-case basis, the information required depends on the GM animal, its GM trait(s), the intended use, and the receiving environments. Previous experience and knowledge with non-GM animals or similar GM animals should also feed into ERA.

### 4.1. Challenges with Regard to the Comparative Assessment of GM Farmed Animals

The comparative assessment of the GM animal with relevant (non-GM) comparators can give indications on the intended effects of the novel GM trait, but also whether it may be linked to unintended effects and, therefore, potential hazards under a range of environmental conditions [11]. The comparative assessment requires an assessment of differences in phenotypic characteristics between the GM animal and its comparator as well as compositional differences in the food and feed produced from the animals [11]. In addition to production-relevant traits, the focus should also be on the assessment of the health and welfare status, e.g., by assessing common parameters such as feed intake, performance (growth and development), feed efficiency, behavior, or reproductive functionality and also clinical chemistry and hematology. As of today, no comprehensive comparative assessment is available from any regulatory authority for the two GM animals discussed in this article, i.e., the slick-haired cattle and growth-enhanced carp (see Section 3).

The phenotypic parameters recommended in the guidance documents should be specifically tailored to the specific type of GM animal and should consider the specific farming conditions. Any observed differences in compositional, phenotypic, or behavioral characteristics between GM and non-GM animals are also of relevance for the assessment of environmental risks. For example, the specific compositional compounds to be assessed in tissues of the GM animals relevant for food/feed use are also of environmental relevance if, e.g., growth-enhanced carp is raised in a polyculture or escapes to natural habitats from ponds and fish farms. However, species-specific parameters, comparable to those used for the comparative assessment of GM crop plants (e.g., at OECD level [69]), still need to be developed. The only available OECD consensus document for a farmed animal addresses Atlantic salmon [70].

With regard to the selection of a non-GM comparator for the GM animal, the respective breeding line must be selected. In fish, the specific fish strain used, and its gene–environment interactions, significantly affect the phenotypic outcome of the genetic modification, as evidenced for transgenic growth-enhanced salmon [54,55]. The use of a non-GM counterpart with the same size or weight, for comparison of GM and non-GM fish as recommended [10], is of limited use for ERA. In fish, size is generally related to age, and growth-enhanced fish will reach their marketable size at an earlier age. Comparing a growth-enhanced and a conventional fish of the same size will compare different age classes and, therefore, developmental stages. While, for the assessment of food safety, the comparison of individuals of similar size is relevant for the commercialization of the fish, for ERA, the specific developmental stage of the fish is of importance, as it affects specific life history traits (e.g., feeding, behavior, etc.) and, consequently, potential adverse environmental effects.

The EFSA recommends the use of non-GM animals with similar characteristics or traits (i.e., a non-GM surrogate animal) as additional comparators, which may be of relevance for the slick-haired cattle [9]. Slick-haired cattle have also been conventionally bred; however, a different breed was used (see Section 2), which may impact the outcome of the comparison due to breed-specific health and behavioral differences unrelated to the genetic modification of the PRLR gene. In addition, the EFSA recommends considering issues with regard to in-breeding and extreme breeding practices leading to hereditary genetic defects with offspring suffering from pain or injury (e.g., for non-food GM animals such as dogs see, e.g., [71]). Also, for cattle, health and welfare issues due to breeding goals that focus on productivity increases are known [72] and require consideration in a comparative assessment of the GM cattle with their non-GM counterparts.

For the ERA of GM plants, a range of non-GM varieties are used as additional comparators, in order to put any observed agronomic or phenotypic differences between the GM plant and its non-GM parental plant into context [73]. For the ERA of GM animals, such an approach is currently not foreseen. The interpretation of observed differences between the GM animal and its conventional counterpart will, therefore, require further guidance as regards the assignment of biological relevance to observed differences with respect to potential hazards for animal health and welfare and for the environment. In addition, normative specifications of harm in animal production need to be disclosed (e.g., for animal health and welfare) or are still to be defined in order to increase the interpretability of the results of the comparative assessment (see also [74]).

### 4.2. Challenges with Regard to the Assessment of Environmental Risks of GM Farmed Animals

#### 4.2.1. Environmental Risks of GM Farmed Animals Are Linked to Their Farming Systems

The individual risk areas outlined in the EFSA guidance for the ERA of GM animals [11] address any changes due to the genetic modification and the resulting potential hazards of the individual GM animal. Many GM traits in GM farmed animals, exemplified by the two case studies, aim at increasing the productivity of the individual animal, e.g., its milk or meat yield (see also [9,12]). The GM animals will, therefore, add to the production efficiency of the specific farming system, either via the reduction in productivity losses (e.g., due to adverse temperature conditions in the case of slick-haired cattle) or by directly increasing productivity (e.g., due to increased feed efficiency in the case of growth-enhanced carp). Any assessments in ERA that are carried out at the level of the individual animal can only address potential effects, which may occur through (intended or unintended) the phenotypic effects of the genetic modification. For example, there are indications that GM growth-enhanced carp have a more efficient protein metabolism [59], thus requiring different fish feed to fully exploit the growth-enhancement trait when raised commercially. GM growth-enhanced fish have a higher feeding motivation, foraging rates, and food competitive behavior with increased feed intake compared to non-GM fish [55]. Therefore, any change in fish feed quality or quantity for this GM fish may contribute to already existing adverse environmental effects of fish aquaculture, such as the release of organic pollutants [75], which would have to be taken into consideration in ERA (see risk areas 6 and 7, Table 1).

**Figure 1:**
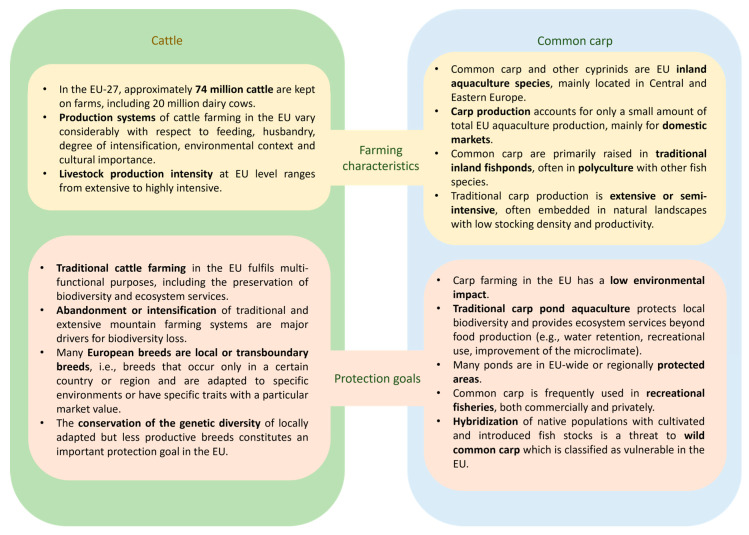
Farming systems and protection goals for cattle [96,97,98,99,100] and common carp [79,101,102,103,104] in the European Union.

However, adverse environmental effects of GM applications of animals for agriculture or aquaculture also have to be considered in the context of the specific production system in which the farmed animal is raised. Biodiversity risks of GM growth-enhanced carp are linked to the type of aquaculture facility where it is raised. In the EU, common carp farming is considered extensive and is mostly conducted in natural ponds, providing a range of ecosystem services (Figure 1). In this type of freshwater aquaculture system, connections of inland facilities with natural water bodies, for freshwater supply or water runoff, enable fish to escape into natural habitats. Accidental escapes are reported from freshwater fish raised in inland fishponds [76,77,78]. Detailed data of escapes of common carp from inland aquaculture facilities are lacking, but it is known that ornamental carp varieties can escape from ponds [79]. For some Asian carp species, escapes from fishponds into natural habitats have been confirmed [80]. Escaped GM fish may be able to establish, persist, and reproduce in natural water bodies that meet their habitat requirements. In addition to its cultivation in outdoor aquaculture facilities and freshwater ponds, carp is an important species for angling and sports fishing [81,82]. Deliberate stocking of natural water bodies for commercial and recreational fisheries with different carp species, including common carp, commonly occur across Europe [83,84,85]. These risks are less likely for GM fish raised in contained freshwater tanks, where organic pollutants, spillover of pathogens, and parasites to native populations, or use of pharmaceuticals and disinfectant, are of environmental relevance [75,86,87].

In the EU, the cattle production sector varies considerably, in terms of production systems, degree of intensification, environmental context, and cultural importance (Figure 1). While intensive farming systems focus on efficiency in production, extensive and traditional farming systems in less favored, e.g., mountainous, areas are multi-functional [88]. Cattle farming systems differ according to feeding and related housing systems, ranging from whole year grazing on pastures to zero-grazing and a high dietary proportion of non-forage feedstuffs, i.e., concentrate [89,90,91].

The ERA of GM farmed animals should consider the differences in the animal production and management systems in which the animals are raised [11]. However, environmental risks that go beyond the individual animal are difficult to address in ERA. For the examples discussed in this article, the abandonment of certain production systems (e.g., extensive cattle farming or traditional carp aquaculture) or their intensification would harm relevant EU protection goals (Figure 1). Environmental risks may also occur due to the extension of animal production to novel environments, implying land use changes that affect biodiversity. Global shifts in cattle farming due to temperature constraints may favor the relocation of slick-haired cattle to environments which are currently not used for cattle farming [45]. The indirect environmental implications that may occur, due to changes in production systems or spatial production patterns of novel biotechnological applications in agriculture, have been recognized in the literature (see, e.g., [92]), but are difficult to assess in an ex-ante ERA approach that focuses on the effects of the individual GM animal. Experience with the assessment of systemic effects of GM plants in herbicide-tolerant cropping systems has shown the importance of the careful selection of alternative non-GM cropping systems for the conclusion on environmental effects of a specific GM crop [93,94]. We propose to address such indirect and systemic effects, including land-use changes, by the selection of representative benchmark production systems (see Section 5) and post-market environmental monitoring, e.g., by use of existing land use monitoring systems in the EU [95]. Contextualizing potential environmental impacts with the specific production system can be combined with addressing the sustainability claims put forward by developers of GM animals with regard to increased efficiency of food production and the resulting lower ecological impacts, e.g., by applying a separate assessment scheme, which takes the respective production system of the GM farmed animal into account (see also Section 5).

#### 4.2.2. Risks to Biodiversity and Nature Conservation Are Likely to Occur Through Stocking or Escape of GM Farmed Animals into Natural Habitats

Biodiversity risks of GM animals used in agriculture and aquaculture are likely to differ between land and aquatic farmed animals (Table 1). Land-farmed animals, like cattle, raised in intensive husbandry systems, are typically under human control in all aspects of their life cycle, including reproduction, feeding, and movement, although contact between domestic cattle on pastures and the European bison has been reported [105]. Aquatic species like common carp are, on the other hand, far less controllable, with a likelihood of escape from production systems into natural habitats and hybridization with wild conspecifics. Consequently, risks to biodiversity and nature conservation are of higher relevance for GM fish than for GM cattle.

A lot of research efforts have been undertaken to address potential environmental risks of transgenic growth-enhanced fish, specifically Atlantic salmon (summarized, e.g., by [55,106,107]). The epistemic uncertainties associated with the environmental risks of GM salmon led to the regulatory decision that restriction and prevention of environmental exposure is necessary [106,108]. In particular, the potential harm to native salmon stocks could not be excluded in ERA and was considered critical. Such risks were also identified for GM sterile Atlantic salmon, which were assessed recently by the Norwegian Scientific Committee for Food and Environment [109]. The GM fish were intended for experimental trials in sea cages in the marine environment. Based on several uncertainties and data gaps, the Scientific Committee concluded that the trials would pose high risks for wild Atlantic salmon populations in Norway.

Similar considerations are relevant for growth-enhanced GM carp. In the case of commercialization, biodiversity risks are evident due to the specific biological characteristics and farming systems of this freshwater species. Farmed fish that were deliberately or accidentally introduced into natural European freshwater habitats have a range of well-documented adverse impacts [110]. Domesticated carp have become feral or even invasive in natural habitats in a range of countries worldwide. In North America, Australia, and New Zealand, common carp and other Asian carp species are classified as invasive or noxious aquatic species. They were imported for use in aquaculture ponds or were accidentally released into natural habitats, e.g., by flooding [111]. In these countries, the successful reproduction of feral carp and adverse ecological impacts thereof have been confirmed [112,113,114]. As domesticated carp can reproduce under natural conditions, impacts on wild, autochthonous common carp stocks through hybridization are likely. Wild, feral, and domesticated strains of common carp can be genetically differentiated [104,115]. Wild populations of common carp exist naturally only in their native range in the Black, Caspian, and Aral Sea basins and adjacent rivers [79]. The wild common carp is classified as vulnerable in Europe according to the assessment of the International Union for Conservation of Nature (IUCN), due to population declines within its native range [79]. The ecological and evolutionary consequences of hybridization events for native fish stocks are well described, e.g., for Atlantic salmon (see, e.g., [55,116]). Interbreeding mostly results in negative outcomes, such as less fit offspring, and can, therefore, drive wild stocks to extinction. Hybridization of wild fish stocks with cultivated and introduced stocks is considered a threat to wild common carp as native populations hybridize readily with morphologically similar non-native strains [104,115]. The genetic diversity and environmental adaptation capacity of wild fish populations is an important protection goal [15].

In addition, common carp is able to hybridize with other cyprinid species such, as gibel carp (*Carassius gibelio*), and produces fertile F1 and viable F2 generations when occurring sympatrically [117,118,119]. Gibel carp is considered invasive in Europe and has been introduced accidentally into fishponds from where it enters other ponds through connecting channels. Adverse effects of invasive gibel carp on other fish populations and community structures through competition are also well documented [120].

The specific GM trait, resulting in a larger body size in GM carp, may have numerous implications for biodiversity and nature conservation at different ecosystem levels if these fish thrive in natural habitats [121]. In short-term experimental assessments with transgenic, growth-enhanced carp, increased body size did not confer an advantage in breeding competitiveness and showed lower juvenile viability [122]. However, the implications for fish communities and biodiversity due to changes in fitness-related traits, such as fish size and growth rate, are complex and need to include effects on predation as well as intra- and interspecific competition [106]. Carp is an omnivorous fish that prefers muddy waters where they churn up the ground when searching for food. Experiments with physiologically growth-enhanced salmon showed increased activity and reduced antipredator behavior, but also changes in habitat use and nutrient excretion [123]. Such competitive interactions for prey or habitat use between larger GM fish and wild fish can have adverse consequences for resource acquisition and, therefore, for the growth and development of wild conspecifics. Larger body size and changed feeding behavior can, therefore, adversely affect aquatic ecosystems [107], further exacerbating biodiversity impacts observed for feral or invasive carp species (see, e.g., [83,124,125]).

### 4.3. Challenges with Regard to the Assessment of Animal Health and Welfare of GM Farmed Animals

As for ERA, the assessment strategy for animal health and welfare is based on a comparative assessment approach of the GM with a non-GM farmed animal [10]. According to the EFSA, the health and welfare of GM animals can be affected by the specific GM trait and may result in changes in management and husbandry due to this trait, reflected by a separate risk area to be addressed in ERA (see Table 1, risk area 7, [10,11]). Such risks may occur, e.g., due to specific (bio)containment measures for GM fish, which may create animal welfare issues [15].

The assessment pertains to the question of whether GM cattle have comparable health and welfare issues as non-GM animals. This can be assessed by evaluating the animal under respective conditions, in the case of slick-haired cattle this would include heat stress. While such an assessment is useful and necessary to evaluate the functionality of the GM trait, it cannot answer the question whether the animal would be used to endure farming conditions similarly to those currently imposed on non-GM cattle [45].

In addition, issues caused by unintended alterations, due to the specific technology applied, also require an assessment with respect to implications for animal health and welfare. Such unintended effects of the genetic modification, i.e., off-target effects or genetic mosaicism, have already been reported for GM cattle [25] and for GM fish [43], and also for other GM animals [13]. Unintended effects in GM animals due to the specific vector construct, such as the accidental introduction of exogenous DNA fragments into the animal’s genome, may lead to the rejection of regulatory approval even without specific assessment of the implications for animal health and welfare [126,127]. For the slick-haired cattle, the consequences of the potential pleiotropic effects of the prolactin receptor truncation for animal health have been discussed recently elsewhere [45]. Pleiotropic effects on the animals’ phenotypes, due to the position of the genetic construct, have been well described for transgenic growth-enhanced salmon [55].

According to current guidance for the ERA of GM animals, not only the health and welfare of the GM animal must be taken into consideration, but also the health and welfare risks of non-GM mammals if they are raised together (Table 1, risk area 8). An assessment of non-GM animal welfare is not required for GM fish, based on the notion that fish like Atlantic salmon are generally raised in monoculture facilities. In the EU, traditional carp farming includes polyculture in ponds, e.g., for the grow-out of carp; therefore, impacts on other fish species raised together with GM carp should be taken into consideration.

Slick-haired cattle will be used in production systems where cattle productivity decreases due to high ambient air temperatures. Heat stress causes declines in cattle productivity in addition to a range of adverse effects on animal health and welfare [128,129,130,131,132]. Currently, farmers use several options and measures to cope with heat in cattle farming. These are mostly based on housing and construction measures, such as ventilation systems, construction of shaded areas, and sprinkler systems. However, additional management measures, such as adaptation of feeding times and feed composition, are also applied (for overview see [131,133]). Welfare issues may, therefore, arise for conventional cattle when raised together with slick-haired cattle, if farmers are reluctant to invest into the technical adaptations needed to alleviate heat stress, e.g., due to cost efficiency, also referred to as “negligence” by [45].

As the farming system, and the breeding line itself, affects the health and welfare of animals for agriculture and aquaculture, e.g., dairy cattle [91], the health and welfare issues of the GM farmed animal should also be addressed in the context of different commercial farming systems and breeding lines used in the EU. For this purpose, the EFSA has suggested a staged assessment approach for the animal welfare assessment, including field trials in representative farm environments, which should reflect the range of systems and managements that are common within the EU [10]. Contextualizing a specific GM farmed animal in its production system faces challenges similar to the assessment of environmental risks (see Section 4.2). In the context of ERA, it will be challenging to only consider the aspects of animal welfare related to the GM trait, while ignoring broader implications, such as challenges relating to animal welfare that already exist within the respective production system.

## 5. Considerations for a Broader Assessment of GM Animals for Agriculture and Aquaculture Beyond ERA

The existing regulatory frameworks for GMOs contain provisions for risk assessment and risk management, to ensure that possible effects on human and animal health, and on the environment, are identified and minimized as best as possible, prior to release or marketing [134,135]. However, emerging biotechnology applications for animals in agriculture and aquaculture raise important questions regarding wider environmental impacts, sustainability aspects, and associated ethical and societal implications [92]. Consequently, a wider assessment and oversight to address the complexities and uncertainties associated with emerging biotechnologies has been recommended [92,136]. Indeed, some legal frameworks, such as the Norwegian Gene Technology Act, require a broader assessment of GM animals that goes beyond health and environmental risk assessment [137]. In addition, the regulatory and guiding frameworks in the EU and at the international level by the Cartagena Protocol on Biosafety also allow addressing broader considerations. The latter frameworks, however, do not provide concrete guidance for the framing of broader governance of technology, nor do they address the scope of considerations and risks that should be taken into account by approaches such as sustainability analysis (SA) or technology assessment (TA).

In the following chapters, we thus discuss how to frame and develop a SA and indicate aspects which are relevant for a TA for GM applications of farmed animals. A similar approach was discussed for GM micro-organisms in a recent review [138].

### 5.1. Sustainability Analysis (SA) of GM Animals for Agriculture and Aquaculture

The transition to a more sustainable and environmentally friendly food and feed production is an important objective of agricultural policies at the international level, the EU, and in many individual countries. In the EU, general policies to increase the sustainability of all agricultural production systems were developed in the context of the European Green Deal and the Farm-to-Fork strategy [139]. Countries, such as Norway, have been forerunners to developing and implementing regulations for sustainability assessments of GMOs, including GM animals, such as GM salmon, for use in aquaculture [137]. The development of an evidence-based framework for sustainability analysis of GM farmed animals would facilitate assessing the claims put forward by different stakeholders, including developers as well as some regulators and scientists, stating that GM animals may contribute to food security and sustainable production [4,18,140]. With regard to the case studies addressed in this article, specific sustainability claims have been made for both GM farmed animals, GM slick-haired cattle as well as GM growth-enhanced carp, such as the following:

- The development of the GM slick-haired cattle is supposed to make milk and beef production more resilient to the effects of rising air temperatures and to mitigate thermal stress on cattle caused by the current and expected increase in global temperature levels [92,141]. Thermal stress is predicted to affect the health and welfare of cattle as well as their productivity, e.g., concerning milk yield in summer but also the overall environmental footprint of cattle production [30,142].
- Growth-enhanced GM fish, including carp, salmon, and other finfish species, have been developed to increase their production efficiency in aquaculture based on increased feed efficiencies, growth rates, and body weights, with potential benefits for competitiveness [109,137]. It has been suggested that such GM fish could also increase the global availability of foods produced from farmed fish and thus be beneficial for food security and the availability of an alternative protein source to meat produced from land-based farmed animals.

The GM animals discussed in the case studies above have not yet been subject of a formal sustainability assessment. However, a number of exploratory studies have already provided considerations for a broader regulatory assessment. These include considerations of sustainability aspects of biotechnology products, including GM animals [92], and a sustainability assessment framework for GM salmon [30,137]. The approaches described by Kuzma et al. [92] for a broader assessment of GM slick-haired cattle, according to the US regulatory framework, and by Blix and Myhr [137] for GM salmon, according to the requirements of the Norwegian Gene Technology Act, were considered for the general approach described below, which is based on the SAFA guidelines [143]. However, due to the different legal backgrounds of both studies (USA and Norway) and the specific aspects related to the intensive salmon farming in saltwater aquaculture as considered by Blix and Myhr [137], these frameworks do not cover all aspects relevant for animal farming in the EU, including freshwater (carp) aquaculture. With a view of the current lack of practical experience with the sustainability analysis of the farming of GM animals in the EU, some basic aspects for such analyses are discussed in the following sections, namely, issues relevant for framing and scoping SAs, as well as the reference systems for comparisons and the relevant issues which should be addressed when performing such studies.

#### 5.1.1. Framing a SA of GM Farmed Animals

In general, a SA of GM farmed animals should cover the three dimensions of sustainability—social, environmental, and economic sustainability—that are typically considered in this context [144], and it should complement issues addressed during the risk assessment of the GM farmed animal in question. These three pillars of sustainability were also used as a reference point in other relevant studies [92,137].

For the SA of GM farmed animals, the existing body of work on sustainability assessments of agriculture and food systems needs to be taken into account. Initial considerations for a comprehensive sustainability assessment framework for food systems were developed by the Joint Research Centre (JRC) of the European Commission [145]. For sustainability assessments of agricultural production, several frameworks are available [146], an example being the SAFA guidelines developed by FAO [143]. The SAFA approach is based on a range of themes supported by a multitude of indicators which can be adapted to fit the assessment requirements for different agricultural production chains in agriculture, forestry, fisheries, or aquaculture [147]. The implementation of the SAFA approach is thus based on the adaptation of its thematic scope to the situation of the assessed entities, e.g., with respect to our case studies, livestock (cattle) farming and freshwater aquaculture (carp production). In a recent study, Paçarada and colleagues [148] recommended the SAFA guidelines to analyze the sustainability of supply chains for dairy products. They identified the SAFA guidelines as an easily accessible tool which integrates multiple sustainability aspects in a comprehensive way and may thus be used to address the sustainability of different production systems with certain adaptations [148]. Also, in the context of commercial fish production, the SAFA tool has already been used, e.g., for the assessment of the production of pangasius fish (*Pangasianodon hypopthalamus*) in Bangladesh, according to a certification scheme for responsibly farmed seafood (Aquaculture Stewardship Council) [149]. Based on these experiences and examples, we conclude that the SAFA guidelines can also be applied to sustainability assessments of GM livestock and fish production systems.

Generally, the SA is based on a different approach than risk assessment as it compares different agricultural or aquaculture production systems with or without the use of the GM farmed animal rather than directly comparing the GM animal with its non-GM counterpart. It thus reflects the complex responses of whole production systems and supply chains to the introduction of (technological) innovations. In addition, the SA of GM farmed animals needs to take the different aspects and management measures relevant for the concerned geographical area into account. Thus, the following discussion concentrates on the respective GM animals used as case studies in European cattle production systems as well as in carp aquaculture systems commonly used in Europe (see Figure 1).

#### 5.1.2. Reference Systems and System Boundaries for the SA of GM Farmed Animals

##### Slick-Haired Cattle

The SA of GM slick-haired cattle should consider five European cattle production systems for dairy as well as beef production with GM cattle or non-GM cattle as reference (Table 2). These production systems are typical for either highly intensive production in confined indoor housing conditions or production at a moderate or low level of intensity, as is common for organic production, including suckler cow production, as the least intensive production system. It has to be considered that, due to the complex interactions between the elements of the production system and factors impacting on them, the specific effect of the GM trait on the whole production system may not be easily identified. Considerations for system boundaries and relevant system elements can be derived from Life Cycle Assessment studies (LCAs); in particular, a LCA designed to assess the effects of technical measures to mitigate heat stress in dairy production systems, as proposed, e.g., by Herzog et al. [133].

##### Growth-Enhanced Carp

In Europe, two different commercial production systems for GM growth-enhanced carp need to be considered: (1) farming in traditional European fishponds; and (2) farming in contained tanks systems (if required for the implementation of containment measures, such as, e.g., for the rearing of GM salmon in Canada, see [40]). These production systems have significantly different characteristics. Aquaculture in a contained system requires a lot of material, energy, and agricultural inputs. For pond production in carp aquaculture systems, biodiversity and ecosystem services based on the recreational and touristic value of such ponds and conservation of traditional practices are important aspects. The traditional non-GM carp pond aquaculture could be used as a reference system. In assessments focusing on commercial fish production, the system boundaries should be defined in a way that all life stages of carp, and the respective production facilities, are covered, as well as relevant inputs (e.g., feed) and outputs (e.g., waste water). In order to assess all relevant aspects of the ecological and social dimensions of sustainability, the assessment of carp production in ponds also has to consider that this type of aquaculture production has significantly shaped the landscape in the respective countries where carp is traditionally farmed. However, new concepts need to be developed to include ecosystem services provided by the respective landscape into sustainability analyses.

In addition to the commercial production of carp in ponds and tanks, the use of carp ponds for recreational purposes (e.g., hiking, swimming, and angling) also has to be addressed, which may challenge defining the system boundaries for this species for a SA. Recreational anglers are currently the predominant users of wild fish stocks occurring in inland waters in the temperate zone [151]. However, the exact amount of fish collected by these anglers may be difficult to estimate [152]. Specifically, in carp fishing, recreational fishers are generally interested in catching single large-sized trophy fish [153], which may favor the use of GM growth-enhanced carp in freshwater lakes used for recreational angling. Stocking of common carp in natural, freshwater bodies can create fishable stocks that would not be present without stocking in aquaculture, e.g., in previously fish-free ponds or heavily harvested water bodies [154]. In addition to intended legal and illegal stocking, the unintended dissemination of farmed or naturally occurring fish has to also be considered.

#### 5.1.3. Issues to Be Addressed in the SA of GM Farmed Animals

The SAFA guidelines provide a basic outline for a set of indicators to evaluate risks and opportunities, as well as to emphasize trade-offs for the sustainable development of animal production [143]. The basic outline covers 21 sustainability themes, divided into 58 sub-themes. However, some sub-/themes are not specific to animal production systems and can either be disregarded, or need to be adapted, to provide suitable indicators. The general approach provided by the SAFA guidelines has been used previously to establish a sustainability assessment framework for European livestock production systems [155,156]. An outline for a SA of animal production systems based on adapted SAFA guidelines is presented in Table 3. It is important to note that aspects considered under the dimensions of ecological integrity and animal health and welfare overlap with aspects that have to be addressed during environmental risk assessment (see discussion in Section 4). We propose that animal health and welfare needs to be considered more prominently in a SA of GM farmed animals, given its importance in the sustainability debate of GM animals [15,16,137]. In the SAFA guidelines, animal welfare is covered as one sub-theme within the ecological dimension, which is neither appropriate in terms of reflecting its uniqueness nor in terms of the diversity of indicators necessary for its comprehensive and valid assessment [143]. For both case studies, animal health and welfare is a cross-cutting issue, and is relevant for environmental risk assessment (see Section 4) as well as in a sustainability assessment.

##### Slick-Haired Cattle

For GM slick-haired cattle production systems, limited data is available concerning the sustainability dimension of ecological integrity [30,141,157]. As put forward by developers, the lower heat stress, as presumably experienced by the GM cattle, results in a higher feed intake rate and a better efficiency with regard to milk production and body mass gain. So far, these claims have, however, not been substantiated by data for GM cattle but inferred from experience with non-GM cattle. Based on this presumed increased efficiency, it is estimated that the ecological impact of GM slick-haired cattle is lower per unit of the relevant product (milk or meat). However, such performance gains may be exploited to increase production intensity, decrease production costs, and, hence, product prices, and could subsequently lead to increased consumption of beef and dairy products. Kuzma et al. [92] noted that an increased production and consumption may potentially lead to a detrimental increase in environmental resources and land usage, especially if production is expanded to areas previously not suitable for cattle farming. This may also lead to adverse effects on the environment and climate change, e.g., due to overgrazing [92], and thereby present a substantial concern from an overall sustainability perspective. Increased productivity could also result in increased absolute resource demands (e.g., feed, water, and inputs such as fertilizers), which, in turn, would increase the climate and environmental impacts associated with the production of these resources (indirect supply chain effects) as well as the direct emissions from cattle farming [158]. Conversely, rising productivity could also reduce resource demands as well as impacts per unit of production (e.g., CO_2_-equivalents or nitrogen emissions into water bodies). This trade-off between absolute figures on the one hand and efficiency gains on the other is complex. GM slick-haired cattle could offer long-term cost advantages, specifically also for subsistence and smallholder farmers if they gain access to the technology. However, the access to the GM slick-hair technology could also pose significant challenges for small-scale farms, e.g., due to high licensing and implementation costs, which could undermine their competitiveness compared to larger farms [158]. The actual impact would heavily depend on the costs associated with the use of this technology as well as on supportive political frameworks and subsidy programs designed to ensure broader accessibility.

##### Growth-Enhanced Carp

Due to the multifunctional uses of traditional carp pond aquaculture in Europe, a sustainability assessment should consider the negative impacts on biodiversity and ecosystem services of GM carp, as identified in ERA, as key aspects of the ecological integrity dimension (see also Section 4). As for ERA, in the SA-context, the preservation of wild stocks, as identified for salmon, is also an important aspect for carp. Specifically, with respect to the multiple ecosystem services of traditional carp production in the EU, certain aspects are not well addressed by the SAFA guidelines, e.g., recreational services. The economic dimension covers aspects such as, e.g., coexistence and freedom of choice for producers and consumers, issues that are also discussed in GM crop production and which are also relevant for GM freshwater fish, like carp. One of these aspects is whether GM carp production negatively affects non-GM aquaculture (particularly with respect to biodiversity, economic resilience, and freedom of choice, Table 3). This may be particularly relevant for organic and certified non-GM fish production but also for areas promoting sustainable recreational angling, which may be affected if GM fish are stocked or accidentally introduced into their angling waters. In the social dimension, specific themes for GM carp aquaculture are food security and healthy food, since aquaculture production is promoted in order to reduce pressure on fish production from marine fisheries. However, trade-offs due to the increased use of fish feed (derived from marine fisheries) which is necessary to achieve higher growth rates for GM growth-enhanced carp, may compromise this aim. Local traditions and the use of local fish strains, which are associated with traditional fishpond landscapes, are also important, and potential impacts on the traditional aquaculture practices have to be considered, which may also affect tourism. Adverse implications on the animal welfare of carp may result from the growth-enhancement trait, such as increased appetite and a greater probability for the occurrence of hunger.

### 5.2. Broader Consideration of Governance Issues Raised by GMA Applications by Technology Assessment (TA)

Technology assessment (TA) strives to assess societal implications of, broadly speaking, emerging technologies. It addresses ecological, economic, cultural, political, legal, or health implications, building upon disciplinary, inter- and transdisciplinary expertise. TA involves, among others, science and technology studies, ecological economics, social ecology, and sustainability science, engaging relevant stakeholders, decision-makers, and various publics. On this basis, TA provides advice to policymakers and society at large, “supporting, strengthening and enhancing reflexivity in all epistemic and social fields of reasoning and decision-making on shaping the scientific and technological advance, on the usage of its outcomes and on dealing with the consequences to present and future society” [159]. In short, similar to sustainability science, TA can serve as an umbrella paradigm, gathering and integrating more specific, disciplinary approaches. De facto, TA often exhibits a stronger focus on societal aspects [160], while sustainability research traditionally comes with a stronger focus on environmental aspects.

To gather the current state of discussion in TA regarding GM animals for agriculture and aquaculture, we performed a review of the literature, researching publications between 2013 and 2023 by ten renowned European TA institutions, regarding TA approaches applied to different biotechnology applications, including GM animals (for details see Eckerstorfer et al. [138]). Our analysis shows that only eight percent of these 135 TA documents focus primarily on the genetic modification of (vertebrate) animals. Additionally, selected animal taxa, and related contexts of genetic modification, are mentioned as examples in about one third of the publications with a generic organismic focus. The discussion of GM animals in TA falls roughly into three categories of application:

- GM animals for biomedical purposes, addressing pigs as organ donors or model organisms, but also “biopharming” (production of biopharmaceuticals in animals) with goats, rabbits, and poultry;
- GM animals for sanitary, human and ecological purposes, applying new genomic techniques, e.g., gene drive approaches, mostly to insect populations and, more specifically, to malaria-transmitting mosquitos;
- GM livestock for food production purposes, for upscaling production rates, disease resistance, and/or for adaptation to (changing or diverse) agricultural environments.

Among the latter, the two case studies selected for the research at hand, GM slick-haired cattle and GM growth-enhanced fish (including one specific mention of carp) were also mentioned. Hornless cattle were another example mentioned several times. Beyond that, species conservation by rendering endangered species fitter, and genetic engineering of companion animals, animals in sports, or silkworms for silk production were also mentioned.

The issues addressed in TA publications on GM organisms cover a wide range of debates, invigorating the role of TA as an umbrella paradigm, combining expertise from environmental risk assessment, sustainability assessment, research into public perception, attitudes and acceptance, research into ethical, legal and social aspects (ELSA) of technology, and research into options for responsible research and innovation (RRI). Risks, ethics, and public attitudes play a significant role in this sample. In TA studies primarily addressing GM animals, ethics, and, specifically, animal welfare play an even bigger role when compared to concerns about other risks. This links to the overall question of which socio-technical innovation is being, or should be, discussed alongside which issues. While agri-biotechnological applications tend to be problematized along risks to human health, the genetic modification of human genomes for research or therapeutic purposes tends to be problematized alongside ethical issues [160]. In fact, the latter discourse (on ethics) is shaped by red lines that are held as absolute givens for a given time, community, and context, but can shift with time and differ between communities, locations, and contexts (see for instance [161,162]). As for the implementation of genetically engineered ‘gene drives’ (genetic modifications designed to spread much more rapidly in a population) to control insect populations and, thus, insect-transmitted diseases like malaria, critical opinions focus on the lack of controllability and irreversibility, resulting in ecological risks and political disagreements [160]. For GM livestock, on the other hand, the focus is on (animal) welfare. Moreover, public attitudes, environmental aspects, agro-technological options, benefits, and regulatory requirements play a role [159,160].

As for the two case studies of GM livestock discussed here, two publications, one published by TA SWISS, a competence center of the Swiss Academies of Arts and Sciences [163] and another published by the Parliamentary Office of Science and Technology (POST) at the Parliament of the United Kingdom in the UK [164], can serve as the most important points of reference. In both reports, animal welfare considerations are a central TA issue. In addition to that, Rapley and Wentworth [164] also address wider implications for the food system, consumer attitudes, public benefits, human health risks, environmental impacts and risks, coexistence with organic farming, trade, and the integrity and dignity of animals. In Lang et al. [163], one dedicated chapter on genome editing in livestock breeding [165] addresses unintended genomic and phenotypic side effects, animal health risks, environmental risks, monitoring and control (especially with aquaculture), and effects on genomic and species diversity, but holds that, at least for some experts, ethical aspects are key when it comes to acceptance. As most important ethical aspects, Hammer and Spök [165] put forward animal welfare, dignity, and integrity. In both publications, genome-editing animals and animal welfare are seen as linked in various, sometimes contradictory, ways. Genome editing can be seen as holding the potential to foster animal welfare (e.g., GM polled cattle or slick-haired cattle) or to threaten animal welfare, e.g., via negative health effects resulting from genome-editing (e.g., Nile tilapia for growth, as mentioned by [164]). But it is also criticized as a “quick-fix” that sidelines other, more effective options to foster animal welfare, like, e.g., via alternative approaches to animal husbandry, and as further exacerbating negative states of industrialized food production. Harm to animal dignity and integrity seems to be linked to phenotypically relevant genetic modifications rather than to other GM traits [165]. Moreover, the issue of instrumentalizing animals within the farming system is brought forward [164], which is closely linked to issues of dignity and integrity. In this context, reference is made to bioethicists who often point to history to inform this debate, such as a systematic review that draws on examples of using animals as “animal machines” [166,167] or the “de-animalization” when changing the cognitive capabilities or nature of animals by genome editing [164].

With its focus on GM ruminants (including slick-haired cattle), Kleter and Sturme [168] outline factors that impact public acceptance (like objectives of GM: improved animal welfare or increased productivity, and trust), but also indirect effects of GM (like encouragement of dense animal stocking in industrial farming systems), concluding (among other aspects) that “farmed animals should not be bred to enhance traits merely so that they may better endure conditions of poor welfare, and farmed animals should not be bred in ways that diminish their inherent capacities to enjoy experiences that constitute a good life. In addition, regulation of farmed animal breeding should consider the wider effects on the organization of food and farming systems and on society in general, in particular, the need to control the potential of innovation to support damaging farming practices” [168]. Mattalia et al. [169] analyze data on the heat load’s impact on the performances of dairy cattle and point out that “a single trait (production, fertility, or udder health separately) cannot summarize the complexity of heat tolerance, and it is important to consider a cow’s physiological abilities together to define the best compromise. Today, the selection of dairy cattle should emphasize traits related to robustness to prepare future generations of cows able to face warmer conditions” [169]. Fischer et al. [170] present a provisional “Room of Acceptance” for GM slick-haired cattle. They sketch a list of possible concerns or parameters of concern (aim and application domain, animal welfare, benefits for food supply created, downstream economic effects, institutional and regulatory responsibility, intrusiveness compared to conventional breeding, necessity for the well-being of society, organism affected, ownership of the techniques and breed, perceived naturalness compared to conventional breeding, risk perception, unforeseen risks and how these are managed, and who benefits from it), and relevant modulating factors (trust in industry, and values held), and indicate for each of them the (surveyed) acceptance performance among experts and stakeholders. “Room of Acceptance” thus reports on the observable multi-dimensional de facto acceptance among various groups, not on deducible ethical acceptability. It is advertised as a “toolbox that helps us have an open conversation about the use of technology in farming, which can help to identify topics where agreement between opponents and supporters can be reached” (for details see [170]).

Alongside all these detailed discussions of potential issues raised by various experts and publics, the overall assessment by the three national TA institutions, and their respective authors, varies to some degree, possibly mirroring different local regulatory discourses and innovation regimes. Rapley and Wentworth [164] from POST take a rather pragmatic stance, balancing welfare issues with potential benefits as well as with the potential downsides of alternatives to GM animals in food production. Lang et al. [163] conclude in their report for TA-SWISS that the perspectives for GM animals are ultimately limited in Switzerland, with strict risk regulation, small-sized breeding programs, few research programs, and the relevant economic actors being aware of averse public attitudes towards GM and low consumer acceptance. Authors affiliated with the Danish Board of Technology (DBT) [168,169,170] also differ considerably in their conclusions, probably due to different disciplinary background, methodologies, and scopes of discussion. Overall, TA publications on GM animals certainly serve to open up discussions on a variety of issues. Based on their accounts of pertinent issues, especially ethical ones and/or a lack of public acceptance, they unanimously advocate for stronger and more systematic interactions among experts, stakeholders, and various publics.

## 6. Conclusions

The assessment of GM animals used in agriculture and aquaculture may be associated with several challenges, with regard to their environmental risks, but also considering specific animal health and welfare aspects, food systems, and ethical issues. Such wider issues increasingly gain importance in the public discourse on GM farmed animals and may affect the acceptance of biotechnological applications in animal production by different actors in the food chain [15]. The application of new genomic techniques in farmed animals is relatively new, and there is little practical experience with regard to the assessment of the potential risks of the respective animal to the environment and biodiversity, human and animal health, animal welfare, or their impacts on sustainability, in the EU or worldwide. Despite the pioneering work of the EFSA with regard to an extensively elaborated ERA guidance for the assessment of environmental risks of GM animals and GM food and feed, the practicability of this guidance has still to be verified. Similarly, assessment frameworks for sustainability or wider societal issues need to be developed for the specific issues posed by biotechnological applications in GM farmed animals.

Based on our analysis of two case studies on GM animals, we find that knowledge on the functionality of the intended modified traits and information on any unintended changes due to the genetic modification in the GM animal is crucial, not only for the assessment of risks to animal health and welfare, but also of the risks to biodiversity and the environment. We also find that the ERA of GM farmed animals faces several challenges. Specifically, we consider the comparative approach unlikely to be fit for purpose, as it fails to contextualize observed effects in a specific production system of the respective GM farmed animal and to assign biological relevance to observed differences between the GM animal and its non-GM comparator. Without further guidance, it will be difficult to separate effects of the GM trait from effects of the production system or the breeding line, impeding a scientifically robust assessment.

Risks to biodiversity and nature conservation are likely to occur for mobile GM animals in aquaculture, with safety hinging considerably on the capability of the specific husbandry and rearing system to prevent the escape of these mobile organisms into natural habitats. In contrast to farmed land animals, the detection of unintended releases of GM aquatic animals, and their later retrievability, are not straightforward. Monitoring and surveillance systems will have to be set up to register potential spread and dispersal into natural habitats and to prevent longer-term ecosystem consequences, e.g., GM growth-enhanced fish. Existing biodiversity monitoring approaches and networks are currently not prepared to cover effects of mobile GM aquatic animals [171].

The analysis shows that animal health and the welfare of GM farmed animals is an overarching issue across ERA, SA, and TA. While ERA can address animal health and welfare implications of the specific GM trait in a specific context, SA and TA consider wider implications in relation to GM farmed animals, such as general paradigms of, and visions for, animal production or the avoidance of breeds with traits detrimental to animal health and welfare, but also the welfare costs that may occur in the development of GM founder animals. The general aim to increase productivity, or to enable production, under adverse farm conditions by use of GM farmed animals without further consideration of animal health, welfare, and dignity are deemed ethically unacceptable [92].

The consideration of the complex interactions between the GM animal phenotype and a vast number of environmental factors in animal production (such as on-site environmental and husbandry conditions, nutrition, disease pressure, or health management) requires the development of approaches that allow for a sound assessment of potential animal health and welfare consequences when introducing GM animals in agriculture and aquaculture production systems. This includes a holistic sustainability assessment, independent from ERA, which provides a minimal set of health, environmental, and socio-economic variables for each type of GM farmed animal. We propose building upon existing technology assessment approaches and sustainability assessment frameworks for biotechnological applications in GM farmed animals.

Regulatory assessment of GMOs is currently discussed at the EU level, particularly by the initiative of the European Commission to deregulate plants developed by targeted mutagenesis or cisgenesis [172]. At present, EU member states have flexibility to decide whether they wish to cultivate GM crops on their territory based on other, e.g., socio-economic, grounds than risk assessment, based on the provisions of Directive (EU) 2015/412. However, the consideration of wider issues of GM farmed animals, including animal health and welfare, and cultural, societal, or ethical issues, in addition to socio-economic aspects (for overview see, e.g., [137]), by use of a holistic or comprehensive assessment is currently not foreseen, but may be necessary to provide for a responsible governance of innovations of biotechnological applications for farmed animals. Suggestions on how to implement such a broad technology assessment during the current regulatory authorization procedure of GMOs in the EU to inform policy decisions have been made [173]. Transparent communication of issues and the associated attention and knowledge gaps will provide robust information for decision-making by risk managers.

## Figures and Tables

**Table 1:** Assessment of environmental risks of slick-haired cattle and growth-enhanced carp, based on risk areas provided by the EFSA [11]. GM = genetically modified; GMA = genetically modified animal; NTO = non-target organism.

No.	Specific Areas of Risks (According to the EFSA [11])	Assessment for Slick-Haired Cattle	Assessment for Growth-Enhanced Carp
1	Persistence/invasiveness/gene transfer to wild and feral relatives.	Likelihood for hybridization of GM cattle with European bison is low as husbandry and breeding are largely under human control.	Hybridization of GM carp with wild carp populations as well as invasive cyprinids; Gene transfer of GM trait to offspring.
2	Horizontal gene transfer (due to introduction of recombinant DNA).	Not relevant as no recombinant DNA.	Not relevant as no recombinant DNA.
3	Potential changes in the susceptibility of GMA to pathogens, infections, or diseases.	Changes in the susceptibility of GM cattle to pathogens, infections or diseases.	Altered interactions between the GM carp and pathogens, infections and diseases.
4	Interaction with target organisms.	Not relevant as no target organism.	No risk area for GM carp.
5	Interaction with NTO (impacts on biotic components and processes).	Effects on different functional groups of NTOs of GM cattle (e.g., gut micro-organisms and endosymbionts).	Effects of GM carp on organisms, ecosystems, and biodiversity, e.g., through changes in consumption, predation, competition, due to hybridization, or through habitat alteration.
6	Interactions with the abiotic environment.	Altered emission of greenhouse gases or pollutants (e.g., ammonia and nitrate).	Altered tolerance to abiotic factors (e.g., oxygen) and effects on the abiotic environment (e.g., physical or chemical characteristics of the habitat).
7	Environmental impacts of the specific techniques used for management of the GMA.	Changes in husbandry and management or intended use of the GMA in novel environments.	Changes in the production system (incl. specific management practices) of GM carp, such as changes in fish diet and feeding due to specific requirements of GM carp. Changes in excretion due to changed protein metabolism.
8	Impacts of the GMA on non-GM animal health and welfare.	Changes in husbandry and management of GM cattle and their effects on non-GM cattle if raised together.	No risk area for GM carp.
9	Impacts on animal and human health.	Risks to farmers and workers; food safety of products from the GMA (e.g., due to differences in animal behavior and food quality)	

**Table 2:** Typical cattle production systems in Europe relevant for the SA of GM slick-haired cattle. PS = production system; TMR = total mixed rations (based on [133,150]).

PS Aspects	A1 High Intensity Dairy PS	A2 Moderate Intensity Dairy PS	B1 High Intensity Beef PS	B2 Moderate Intensity Beef PS	B3 Suckler Cow PS
Typical housing systems	Confined all year round, mainly loose-house systems *; typically without pasture	Partially confined, mainly loose-house systems **, typically with pasture (summer half-year)	Confined all year round, mainly loose-house systems with slatted floor; without pasture	Partially confined, loose-house systems; typically with pasture (summer half-year)	Partially confined, mainly loose-house systems; typically with extensive pasturing (summer half-year)
Level of performance	>7000 kg milk per cow and year	<7000 kg milk per cow and year	>1000 g body mass gain per day	<1000 g body mass gain per day	<1000 g body mass gain per day
Feeding (type of diet)	TMR, maize and grass silage, concentrates, hay	maize and grass silages (partially TMR), pasture, hay, concentrates	TMR, maize and grass silage, concentrate, hay	maize and grass silages, pasture, concentrate, hay	maize and grass silages, pasture, hay, concentrate

* Partially tied systems in alpine countries. ** Partially tied systems.

**Table 3:** Sustainability dimensions and themes for a SA according to the SAFA guidelines, as well as relevant subthemes for both case studies. GM: genetically modified; GHG: greenhouse gas, tbd: to be determined.

Sustainability Dimension *	Sustainability Themes *	GM Slick-Haired Cattle	GM Growth-Enhanced Carp
Ecological integrity	Atmosphere	GHG emissions, air pollutants	GHG emissions
	Water	Water withdrawal, water quality	Water quality
	Soil	Soil quality, land degradation	Soil quality, land degradation (feed- and pond-related)
	Biodiversity	Ecosystem, species and genetic diversity (e.g., more genetically uniform breeds)	Ecosystem, species and genetic diversity (e.g., preservation of wild stocks)
	Material and Energy	Feed and energy use, waste reduction	Feed and energy use, waste reduction
Economic resilience and efficiency	Production efficiency	Economic viability, volume of production	Economic viability, volume of production
	Economic resilience	(Stability of) net income Costs of production	Costs, increased independence from imported fish products, viability of non-GM carp production
Social sustainability	Decent Livelihood	Job creation, farm income	Job creation, farm income
	Fair trading practices	tbd	tbd
	Equality, non-discrimination, gender equality, vulnerable groups	Freedom of choice for non GM products	Freedom of choice for non GM products
	Human health and safety	Nutrition	Nutrition
	Good governance	Compliance with climate and agricultural policies	Compliance with policies for sustainable aquaculture
	Absence of hunger and thirst	Impact on food security	Impact on food security
Animal Health and Welfare	Comfort	Thermal, physical, (when resting and during locomotion)	Potential occurrence of hunger in GM fish; stocking density
	Good human–animal relationship	Human–animal relationship	tbd

* According to FAO [143].

## Data Availability

No new data were created or analyzed in this study. Data sharing is not applicable.
